# Asbestos Disease Initiation

**DOI:** 10.1007/s11538-026-01605-7

**Published:** 2026-02-19

**Authors:** Alisa DeStefano, Clyde Martin, Dorothy Wallace

**Affiliations:** 1 Department of Mathematics and Computer Science, College of the Holy Cross, 1 College St, Worcester, MA 01610 USA; 2 Department of Mathematics and Statistics, Texas Tech University, 1108 Memorial Circle, Lubbock, TX 79409 USA; 3 Department of Mathematics, Dartmouth College, 27 Main St, Hanover, NH 03755 USA

**Keywords:** Asbestos exposure, Mesothelioma, Pleural plaque, Lung fibrosis, Asbestosis

## Abstract

A model is developed of disease initiation and progression for asbestos related plaque deposition and mesothelioma based on prior models of wound healing and asbestos transport. The model includes short term processes such as macrophage polarization and immune system response, extending the time frame to that of detectable disease. Model results were in a biologically reasonable range. Cancer development and plaque deposition are shown to be dependent on total exposure (intensity * duration). Plaque deposition was sensitive to many systemic parameters, suggesting that assays could be developed to identify individuals with higher risk. Tumor development was sensitive only to a few parameters describing exposure and T-cell levels, indicating potential for immune system intervention.

## Introduction

Mesothelioma is a cancer of the mesothelium, a thin stretchy tissue surrounding the lungs and pleura. It is associated with asbestos inhalation, an occupational hazard for asbestos workers and others in environments with high asbestos. Incidence in the U.S. of this deadly cancer peaked in the 1970’s, declining as extraction and usage shifted to a less toxic form (Weill et al. 2004). The mean latency period (from first asbestos exposure to clinical manifestation of tumor) is 30 years (Weill et al. 2004). High rates of mesothelioma are associated with specific minerals (amphibole, crocidolite) and with fiber length as longer fibers tend to remain in tissue (Barlow et al. 2017; Dodson et al. 2007). Longer fibers are more visible upon autopsy, however, and so this inference may change as new imaging or other techniques become available. Mesothelioma patients may also have lung fibrosis (scarring of lung tissue) and pleural plaques (localized thickening of the pleura, the lining of the lungs).

It is hypothesized that a cascade of processes leading to mesothelioma derives from reactive oxygen species (ROS) released from asbestos fibers (Manning et al. 2002; Shukla et al. 2003). Sources describing the chemistry of asbestos, however, describe asbestos fibers as “basically chemically inert, or nearly so. They do not evaporate, dissolve, burn, or undergo significant reactions with most chemicals.” (Asbestos 2001). It has been found that mobilization of Fe from asbestos fibers requires a chelator and is dependent on pH and temperature (Lund and Aust 1990). Within the cell there are low molecular-weight (LMW) chelators present at millimolar concentrations, which could in principle mobilize iron from the surface of a fiber piercing the surface of the cell (Aust et al. 2011). As intracellular chelation occurs, the iron content of a fiber-containing cell changes. Because the process occurs on the surface of the fiber and dissolution may be slow, iron may be removed over a long period (Aust et al. 2011). In addition, ROS may be formed near the surface of a fiber due to reactions catalyzed by iron (Kamp et al. 1992). The interaction of cells with ROS has been more thoroughly described (Shukla et al. 2003) and is used in this model as the cause of genomic change.

Inflammatory processes associated with asbestos inhalation are similar to those of wound healing, and give a quick response to the presence of fibers, representing a short time frame. Most asbestos workers are exposed for several years, a medium time frame. However, the 30 year period between first exposure and clinical disease presents an interesting puzzle. Short term processes of transport can be coupled with medium term exposure to yield a model producing reasonable estimates of long term asbestos burden in tissue (DeStefano et al. 2021). A similar approach is used here to study disease processes producing plaque deposition, cancer development, and cancer growth. The model developed here is based on various hypotheses in the literature about the mechanisms contributing to mesothelioma and plaques/fibrosis during that latent period and is used to answer questions about the role of exposure level and duration, and immune system “strength”.

## Background

Mesothelial cells ingest asbestos fibers rapidly in a dose dependent manner (Broaddus et al. 1996). Cells cultured in monolayer and flooded with fibers ingested, on average, 4 fibers per cell within 24 hours, at which point $18\%$ were undergoing apoptosis and another $10\%$ necrosis or late apoptosis (Broaddus et al. 1996). However, it is likely that, by 24 hours at these concentrations, more than $50\%$ are dead or dying, indicating a much higher contribution from necrosis (Yang et al. 2010). When a cell receives sufficient damage or is no longer needed, it will normally undergo apoptosis, an orderly process that does not result in production of inflammatory signals or initiate inflammatory processes. Other forms of cell death are necrotic and can trigger an inflammatory response and processes (Yang et al. 2010). The protein HMBG1, released by damaged or dying cells into the surrounding environment, triggers inflammatory responses and marks the cell for destruction (Kim et al. 2019). These responses can cause damage to surrounding cells, tissues, organs or become systemic. There are several types of necrotic cell death. Some types are termed “Programmed Necrosis” by some researchers to emphasize that necrotic death is not a completely uncontrolled cellular explosion but involves various signaling pathways (Kim et al. 2019).

Note that 4 fibers per cell represents an extremely high dose, as autopsy measurements suggest a prevalence of 20,000 to 2,000,000 fibers per gram of lung (Dodson et al. 2007). Estimating $10^8$ cells per gram suggests at most 2 fibers per 100 cells, and at least 2 fibers per 10,000 cells (Wilson et al. 2003). These observations strongly suggest that the fibers removed by macrophages and transported elsewhere, as modeled in DeStefano et al. (2021), may have been first ingested by a mesothelial cell which died, and the whole ensemble transported by the macrophage. This process would create micro-injuries of missing cells all over the mesothelium. The active release of HMBG1 by these dying cells signals release of TNF-$\alpha$ by macrophages, as well as other pro-inflammatory cytokines (Kim et al. 2019).

Successful wound healing is a multistage process involving chemoattractant signals that draw fibroblasts that lay a substrate over which epithelial cells may proliferate and close the wound. As wound closure proceeds, the substrate is removed by an enzyme and the wound tightens against the elastic tissue (Stadelmann et al. 1998). Importantly, cell proliferation comes to a close when the wound is healed. All of these transitions are orchestrated by macrophages and the signals they produce and to which they respond (Snyder 2016). Mesothelial cells dead from asbestos ingestion represent a different type of wound, which is distributed across the tissue. Healing is a diffuse process occurring throughout the tissue surface, rather than at the edges of a macroscopic wound (Mutsaers 2002; Whitaker and Papadimitriou 1985). The process still involves a fibrin substrate and is mediated by macrophages (Whitaker and Papadimitriou 1985).

Macrophages, which are present in all tissues, actively engulf and attempt to remove fibers from tissue, or engulf fibers encased in dead mesothelial cells. Incomplete (frustrated) phagocytosis results in macrophages trapped in tissue with fibers partially inside them, and therefore are likely to account for most of the intra-cellular fiber (Kamp et al. 1992). Macrophages are involved with iron homeostasis in the body and exhibit a range of phenotypes with two extremes, labeled M1 and M2 (Mertens et al. 2016). The cytokine, IL-10, is known to polarize macrophages to the M2 phenotype, while other endotoxin/cytokine combinations are known to promote the “classical” M1 phenotype (Mertens et al. 2016). These molecules are produced by a range of cell types and are part of the “crosstalk” of the immune system. The systems are loosely described as “pro-inflammatory” (M1 and associated signals) and “anti-inflammatory” (M2 and associated signals). Each phenotype is the source of signaling molecules affecting the wound healing process. At one extreme, the M1 phenotype generally occurs in conjunction with inflammation and sequesters iron, creating local anemia that depresses pathogens. At the other extreme, the M2 phenotype releases iron into the tissue, creating conditions that favor cell regeneration (Mertens et al. 2016). Asbestos particles trapped via frustrated phagocytosis become coated with minerals, and are termed “ferruginous bodies” due to high iron content (Ghio et al. 2023). These are believed to produce reactive oxygen species (Goodglick and Kane 1986; Hansen and Mossman 1987; Shukla et al. 2003). Reactive oxygen species are implicated in the initiation of mesothelioma (Shukla et al. 2003).

Fibroblasts are cells that produce molecules comprising the structural matrix of the body, including collagen and scar tissue (Genetics Home Reference 2014).

Mesothelial cells that have ingested asbestos produce a chemoattractant to fibroblasts, as well as fibronectin, a glycoprotein important for wound healing (Bonnans et al. 2014; Kuwahara et al. 1994). These cells then die and are removed by macrophages. Fibroblasts respond to injury by proliferating in response to signals to become myo-fibroblasts, and then laying down a collagen extra-cellular matrix. This process is balanced by dissolution of the matrix by an enzyme. We will assume these processes, which are well described for skin injury (Stadelmann et al. 1998), take place as well in the mesothelium. When the injury is healed the fibroblasts should leave or undergo apoptosis (Genetics Home Reference 2014), presumably in response to some signal. If excessive collagen is deposited there will be scar tissue in the case of skin wounds, or fibrosis or plaque in the case of injury to the mesothelium (Rapini Ronald et al. 2007).

Mesothelial cells (M) and mesothelial cells near fibers (MNF) undergo mesothelial-to-mesenchymal transitions (MMT) to mesenchymal form (MM, MMNF), which allows them to participate in tissue regeneration and is implicated in the onset of mesothelioma (Turini et al. 2019). This transition is dependent on signals largely due to macrophage dynamics. The corresponding process for epithelial cells (epithelial to mesenchymal transition, EMT) is associated with the development of cancer. In both MMT and EMT the mesenchymal form is mobile, loses cell-cell adhesion, and may proliferate. These qualities are necessary for tissue regeneration but are also hallmarks of cancer cells.

Signaling molecules involved in wound healing are generally classed as pro-inflammatory or anti-inflammatory. Pro-inflammatory signals favor macrophage polarization to M1, and are also produced by M1 macrophages. Instead of using the full suite of signalling molecules in the model, one is chosen as a proxy, IL-1 (IL1). Similarly, anti-inflammatory signals promote M2 and are represented in the model here as IL-10 (ILten), although there are many. In addition, tumor necrosis factor alpha (TNF) and transitional growth factor beta (TGF) are separately included, as their roles have distinct functions in the tissue repair process.

Taken together, these processes represent a complex system that could potentially be disrupted by the presence of asbestos fibers, leading to plaque and fibrosis, mesothelioma, or both. The model in this study is used to understand how different exposure conditions could create a range of disease.

## Model Development

**Fig. 1:**
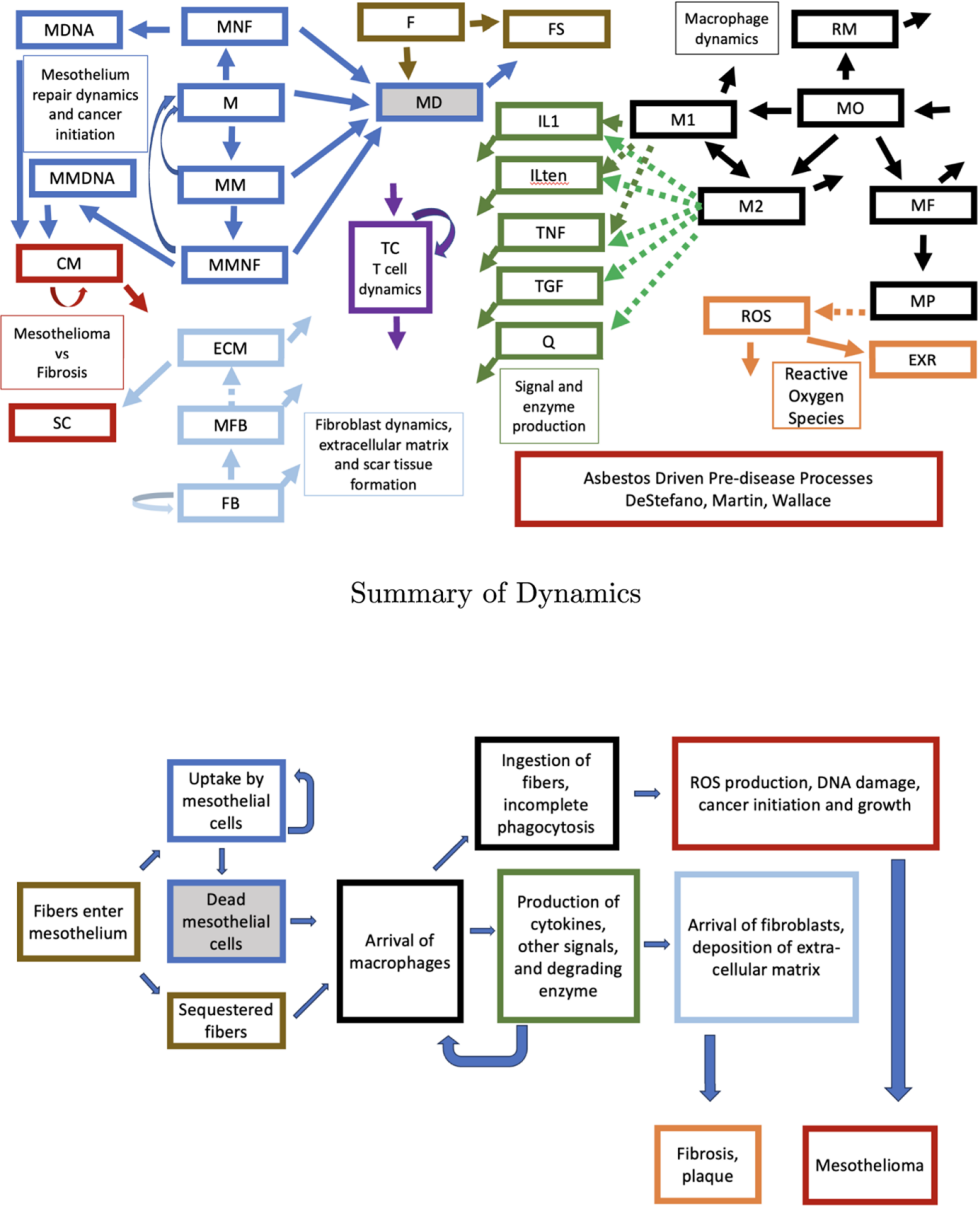
The full compartment model is followed by a simplified version highlighting the key biological processes and outcomes

A visual overview of the model is given in Figure 1.

The general recruitment and polarization process for macrophages is adapted from (Wermuth and Jimenez 2015). Signal dynamics are adapted from (Voropaeva and Bayadilov 2020). The version of fibrosis dynamics in this model is based on a model of Hao et al. (2015) which examines the origin of fibrosis due to abnormal tissue repair.

The model proposed assumes that free fibers in tissue are quickly taken up by mesothelial cells which then die, and fibers inside dead cells are removed by macrophages. It also recognizes that some fibers may get lodged in tissue without being ingested, as uncoated fibers are found in autopsy (Dodson et al. 2007). All tissues contain a population of resident macrophages (RM) derived from circulating monocytes (MO). Depending on the rate of asbestos exposure, some of these will ingest fibers previously ingested by mesothelial cells (MD). Some of those will get trapped in the mesothelium, unable to remove a fiber. Resident macrophages (RM) are routinely replaced by recruited monocytes (MO), and may ingest fibers assumed to be in dead mesothelial cells (MF).

Macrophages are recruited and polarized to type M1 in response to pro-inflammatory signals. After ingesting a fiber they leave. Some are lodged in tissue due to incomplete phagocytosis, resulting in coated fibers remaining in tissue. Macrophages may also be polarized to type M2 depending on signals. The model below recognizes several forms of macrophages: resident macrophages (RM), polarized to M1 (M1) or M2 (M2), macrophages near fibers (MNF), and macrophages with incomplete phagocytosis (MP). The model includes equations for these quantities as well as equations for signals TNF-$\alpha$ (TNF), TGF-$\beta$ (TGF), IL-1 (IL1) (a proxy for pro-inflammatory signals) and IL-10 (ILten) (a proxy for anti-inflammatory signals).

The signals play a complex role in macrophage polarization, mesothelial to mesenchymal transition, summoning of fibroblasts (FB) and transition to myofibroblasts (MFB).

Circulating monocytes (MO) arrive in tissue at a steady rate which is increased in the presence of pro-inflammatory signals. They may also be polarized by signals to M1 (pro-inflammatory signals, PIS) or M2 (anti-inflammatory signals, AIS) and some die. We will represent polarization as the product of a rate of polarization (A) and the probability *p* of choosing polarization M1 ($0< p < 1$). Both A and p are dependent on signals. Let S be the sum of all signaling molecules in the system, including sequestered fibers (FS) that should provoke inflammation, S= (TNF + IL1 + Ilten + FS). It is assumed that TGF plays little to no role in initial inflammation.

We set A= 0.001*kSS* S/(kS + S), allowing a maximum polarization rate 0.001*kSS that increases with overall signal concentration. We then split the polarization according to the prevalence of signals that promote M1 (TNF and IL1), versus those promoting M2 (p and p2 respectively). Note that a suite of signaling molecules is actually involved, but we are simplifying by just using the pro and anti inflammatory signals in the model as proxies for this far more complicated interaction. This leads to auxiliary equations:

S is the sum of all pro-inflammatory and anti-inflammatory signals:

$$\begin{aligned} S= TNF +IL1 +ILten + FS \end{aligned}$$

*A* is the rate of polarization with max 0.001*kSS and half saturation kS:

$$\begin{aligned} A=0.001*kSS*\frac{S}{(kS+S)} \end{aligned}$$

Let *p* be the probability of choosing polarization M1 in the presence of pro-inflammatory signals:

$$\begin{aligned} p=\frac{(TNF+IL1 + FS)}{(kS+S)} \end{aligned}$$

Let *p*2 be the complimentary probability of choosing polarization M2 in the presence of anti-inflammatory signals:

$$\begin{aligned} p2=\frac{(ILten)}{(kS+S)} \end{aligned}$$

The rate of change of resident macrophages, *RM*, = recruitment from monocytes – fiber ingestion – clearance

1 $$\begin{aligned} \frac{d}{dt}(RM)= kRM*MO- \frac{kF*MD*RM}{(qao+MD)} - cRM*RM \end{aligned}$$

The rate of change of monocytes (MO), =normal recruitment – polarization to M1 – polarization to M2 – replacement of RM - clearance

2 $$\begin{aligned} \frac{d}{dt}(MO)= rM - A*p*MO - A*(p2)*MO -kRM*MO -cM*MO \end{aligned}$$

Both RM and M1 macrophages will ingest fibers previously ingested by mesothelial cells in a dose dependent manner (depending on dead mesothelial cells (MD), which in turn depends on fiber inhalation rate) becoming macrophages containing fibers (MF). After fiber ingestion, macrophages remove the fiber and themselves if possible, otherwise they will die and remain in the tissue due to incomplete phagocytosis.

The rate of change of MF, = fiber ingestion rate– removal

3 $$\begin{aligned} \frac{d}{dt}(MF) = \frac{kF*(RM+M1)*MD}{(qao+MD)} - cMF*MF \end{aligned}$$

When macrophages ingest a fiber a fraction of them may experience incomplete phagocytosis (MP), which will kill them while still in the mesothelial tissue. These are thought to be active producers of ROS and interact with the larger model.

4 $$\begin{aligned} \frac{d}{dt}(MP) = k62*cMF*MF \end{aligned}$$

M1 polarized macrophages (M1) develop from circulating monocytes (MO), in the presence of pro-inflammatory signaling (A*p), from M2 to M1 polarization (dependent on signals) and may be lost by M1 to M2 polarization. We assume the polarization between M1 and M2 exhibit the same dependence on signal as that of M. M1 polarized macrophages may also ingest fiber that has been previously taken up by a mesothelial cell that then died, moving to the MF compartment, or may be removed by clearance.

The rate of change of M1 = polarization from MO + polarization M2 to M1– polarization M1 to M2 – ingestion of fiber – clearance

5 $$\begin{aligned} \frac{d}{dt}(M1) = A*p*MO - A*(p2)*M1 + A*p*M2 - \frac{kF*M1*MD}{(qao+MD)} - k29*M1 \end{aligned}$$

M2 polarized macrophages (M2) develop from MO in the presence of AIS signaling, from M1 to M2 polarization (dependent on signals) and may be lost by M1 to M2 polarization or death. We assume the polarization between M1 and M2 exhibit the same dependence on signal as that of M.

The rate of change of *M*2 = polarization from MO + polarization M1 to M2 - polarization M2 to M1 - clearance

6 $$\begin{aligned} \frac{d}{dt}(M2)= A*(p2)*MO + A*(p2)*M1 -A*p*M2 -k29*M2 \end{aligned}$$

Tumor growth factor TGF-$\beta$ (TGF) is implicated in the production of IL-10 and TNF-alpha, as well as potentially playing a role in the larger model. It is produced by both macrophage polarizations (Voropaeva and Bayadilov 2020). However, several review articles have indicated that it is primarily produced by M2, although whether it is produced in response to a signal is unclear (Assoian et al. 1987; Mosser and Edwards 2008; Sharifi et al. 2019). We therefore modify the term in Voropaeva and Bayadilov (2020) as follows:

The rate of change of *TGF* = production by M2 – clearance

7 $$\begin{aligned} \frac{d}{dt}(TGF)= k46*M2 - k48*TGF \end{aligned}$$

Pro-inflammatory signals (TNF-$\alpha$, IL-1$\beta$) may be produced by M1 cells or added by compartments external to the macrophage submodel, but included in the larger model. In particular, HMGB1 is a protein released upon the death of a mesothelial cell (MD), which induces macrophages to release TNF-$\alpha$ (Gaudino et al. 2014). All signals are eventually removed by lymphatic flow. It is assumed that signals removed from solution by uptake via binding to cells are a negligible effect.

TNF-$\alpha$ dynamics (TNF) are summarized as follows:

The rate of change of *TNF* = production by M1 + production by M2 + production by RM – removal

8 $$\begin{aligned} \frac{d}{dt}(TNF)= \frac{cp*(M1+M2+RM)*MD}{(kTNF+MD)} - k42*TNF \end{aligned}$$

The interleukin IL-1-$\beta$ dynamics (IL1) are fit to data in Voropaeva and Bayadilov (2020) and it was found that both M1 and M2 produced this although M1 production was suppressed by high levels of TGF-$\beta$ (TGF) and IL-10 (ILten). Here we rely on the general rule that pro-inflammatory signals are produced mostly by M1, and the anti-inflammatory signal Ilten is used as a proxy for both sources of signal suppression.

The simplified term is given thus: the rate of change of IL1 = production by M1– clearance

9 $$\begin{aligned} \frac{d}{dt}(IL1)= \frac{k58*M1}{(k59+ILten) } - k85*IL1 \end{aligned}$$

For the dynamics of the anti-inflammatory signal interleukin IL-10 (ILten) a model of signals involved in wound healing was fit to data and concluded that IL-10 is produced by M1 in response to TGF-$\beta$ and by M2 in response to low levels of IL-10 (Maeda et al. 1995; Voropaeva and Bayadilov 2020).

The rate of change of ILten = production by M1 + production by M2 – clearance

10 $$\begin{aligned} \frac{d}{dt}(ILten)= \frac{k81*TGF*M1}{(k82+TGF)}+ \frac{k83*M2}{(k84+ILten)}-k85*ILten \end{aligned}$$

Healthy, unexposed mesothelial cells (M) become exposed when near a fiber (F). They will uptake uncoated asbestos fibers (F) and die, creating a population of dead mesothelial cells then removed by macrophages. Under certain signaling conditions they may transition to mesenchymal form (MM). Several studies implicate TGF-$\beta$ in this transition (Lee and Ha 2007; Voropaeva and Bayadilov 2020; Turini et al. 2019). These cells are replaced by the action of MM and MMNF, in response to a multitude of signals. We assume regeneration from both M and MM, which depends on the presence of TGF, and ceases in response to a carrying capacity.

The rate of change of M = - death due to fiber uptake - transition to mesenchymal form + transition from mesenchymal form + regeneration

11 $$\begin{aligned} \frac{d}{dt}(M)=&-wdF*max \left( min(F,M)*\frac{M}{(M+MM)},0\right) \nonumber \\&-\frac{q1*(M*TGF-cFB*MM)}{(s1+TGF)} \nonumber \\&+q3*(MM+MMNF)*(1-M/Cm)*\frac{TGF}{(s1+TGF)} \end{aligned}$$

Mesothelial cells in mesenchymal form (MM) are assumed to behave similarly in response to fibers and fiber exposure. However, in the presence of signals they may also proliferate, likely in response to TGF-$\beta$. We assume that they do so in order to replace M rather than produce more MM.

The rate of change of MM = transition to and from M - death due to fiber uptake

12 $$\begin{aligned} \frac{d}{dt}(MM) =&\frac{q1*(M*TGF-cFB*MM)}{(s1+TGF)}\nonumber \\&-wdF*max \left( \frac{min(F,MM)*MM}{(M+MM)},0\right) \end{aligned}$$

Mesothelial cells near fibers (MNF) increase as fibers enter and are engulfed by macrophages or can die by programmed necrosis in response to ROS. After long exposure to ROS, with cumulative exposure EXR, they may develop DNA damage. They may also polarize to mesenchymal form (MMF) in the presence of signals. The rate of becoming near fibers is chosen to be the same as the rate of death. Upon sufficient cumulative exposure these cells pass to compartments representing cells with DNA damage.

The rate of change of MNF = increase due to proximity of M to fiber - death due to fiber uptake – DNA damage

13 $$\begin{aligned} \frac{d}{dt}(MNF) =&wdF*max \left( \frac{min(F,M)*M}{(M+MM)},0\right) - c2*MNF \nonumber \\&-h2*EXR*MNF \end{aligned}$$

For the mesenchymal form near coated fibers (MMNF) with proliferation we get a similar equation.

The rate of change of MMNF = increase due proximity of MM to fiber + proliferation - death due to fiber uptake - dna damage

14 $$\begin{aligned} \frac{d}{dt}(MMNF) =&- c2*MMNF + wdF*max\left( \frac{min(F,MM)*MM}{(M+MM)},0\right) \nonumber \\&-h2*EXR*MMNF \end{aligned}$$

Mesothelial cells engulf fibers and then die an early necrotic death (dead cells, MD), which interacts with macrophages and signal production. They are cleared by macrophage removal. Mesothelial cells ingesting fiber are assumed to not survive.

The rate of change of MD = increase due to ingestion – macrophage uptake

15 $$\begin{aligned} \frac{d}{dt}(MD)=c2*(MNF+MMNF)*F -\frac{kF*(RM+M1)*MD}{(qao+MD)} \end{aligned}$$

Older cells from both MNF and MMNF populations might develop DNA damage, and might die from the action of T cells (TC). After sufficient cumulative exposure such cells may develop into cancer. Note that we must include the process by which the immune system sends killer cells to remove damaged cells.

The rate of change of MDNA = development of DNA damage – loss due to T cells (TC) – development of cancer

16 $$\begin{aligned} \frac{d}{dt}(MDNA) =&h2*EXR*MNF -kTC*MDNA*TC \nonumber \\&- nM*EXR*MDNA \end{aligned}$$

Similar equations hold for the mesenchymal form MMDNA which we can assume is no longer proliferating:

The rate of change of MMDNA= development of DNA damage – loss due to T cells (TC) – development of cancer

17 $$\begin{aligned} \frac{d}{dt}(MMDNA) =&h2*EXR*MMNF -kTC*MMDNA*TC \nonumber \\&- nMM*EXR*MMDNA \end{aligned}$$

A fraction of these DNA damaged cells may become cancerous (CM), leading to mesothelioma. It is likely that the rate is higher for those in mesenchymal form. Some cancerous cells would be removed by T cells that have been trained to recognize them (TC). These would be a small fraction of all T cells. Also, cancer cells proliferate.

The rate of change of CM = development from DNA damaged mesothelial cells of both forms – death from T-cell activity + proliferation

18 $$\begin{aligned} \frac{d}{dt}(CM) =&nM*EXR*MDNA + nMM*EXR*MMDNA \nonumber \\&- kTC*CM*TC + pC*CM \end{aligned}$$

Free fibers in tissue (F) arrive by inhalation. Many are translocated to other parts of the body. The model assumes the ones entering the mesothelium are taken up by mesothelial cells. Some become lodged in tissue (F) without being ingested.

The rate of change of *F* = fiber arrival to the mesothelium - uptake by mesothelial cells - clearance of fibers

19 $$\begin{aligned} \frac{d}{dt}(F)= I*(heaviside( EX -t)) -c2*(MNF+MMNF)*F -q*F \end{aligned}$$

Reactive oxygen species (ROS) are said to be produced by ingested fibers in macrophages with incomplete phagocytosis (MP), also called ferruginous bodies, and are cleared by the lymphatic system (Dixon and Stockwell 2014; Shukla et al. 2003).

The rate of change of *ROS* = production by ingested fibers - clearance

20 $$\begin{aligned} \frac{d}{dt}(ROS)= hROS*MP -cROS*ROS \end{aligned}$$

Cumulative exposure at time t (EXR), is computed as

21 $$\begin{aligned} \frac{d}{dt}(EXR) = ROS \end{aligned}$$

T cells and natural killer cells (TC) are responsible for identifying and killing cells with DNA damage, whether cancerous or not. They are recruited in response to signals and may also proliferate in response to signals. While recruitment is favored by pro-inflammatory signals (TNF-$\alpha$ and IL1 to name the two in this model), recruitment is suppressed by anti-inflammatory signals (TGF-$\beta$ and ILten in this model) (Adams and Rlloyd 1997). Proliferation appears to follow a similar pattern in infectious disease (Scheurich et al. 1987; Sieling et al. 1993; Su et al. 1991). Similar studies were not found for mesothelioma and T cells are assumed to rise with the prevalence of cancer cells.

The rate of change of TC = recruitment + proliferation – clearance

22 $$\begin{aligned} \frac{d}{dt}(TC)=&\left( \frac{q4*(TNF*IL1)}{(sTGF+TGF)*(sILten+ILten)} +q5*TC*CM \right) *\left( 1-\frac{TC}{k60}\right) \nonumber \\&-cTC*TC \end{aligned}$$

Hao et al’s model of fibrosis tracks macrophages M1 and M2, TGF-$\beta$, TNF-$\alpha$, and IL13, quantities already in the model if we assume IL13 is readily available or tracks with TGF-beta (Hao et al. 2015). We make a few changes, replacing epithelial cells and activated epithelial cells with mesothelial cells (M) and EMT mesothelials cells (MM), and PDGF by dead mesothelial cells (MD). We remove diffusion terms. We make the simplifying assumption that inhibition of matrix degrading enzyme is constant and ignore the role of IL13. This leaves equations for fibroblasts (FB), myofibroblasts (MFB), extra cellular matrix (ECM), scar tissue created in response to ECM above a threshold which we will interpret as plaque or fibrosis (SC), and degrading enzyme (Q), as follows:

The rate of change of FB= background input increased due to pro-inflammatory signals - transition to MFB - clearance

23 $$\begin{aligned} \frac{d}{dt}(FB) = IFB*p -\frac{pFB*TGF}{(TGF+kTGFFB)}*FB- c1*FB \end{aligned}$$

The rate of change of MFB = transition to MFB from FB – clearance

24 $$\begin{aligned} \frac{d}{dt}(MFB)= \frac{pFB*TGF}{(TGF+kTGFFB)}*FB- c1*MFB \end{aligned}$$

The rate of change of ECM= production by MFB in presence of signal (TGF) – degradation due to enzyme Q

25 $$\begin{aligned} \frac{d}{dt}(ECM)=&c2ECMMFB*\left( \frac{TGF}{kFBTGF+TGF}\right) *MFB \nonumber \\&-wdECMQ*Q*ECM \end{aligned}$$

The rate of change of SC = proportional to excess ECM

26 $$\begin{aligned} \frac{d}{dt}(SC)= cSC*MFB*(max(0,ECM-th)) \end{aligned}$$

The rate of change of Q = production by M2 – removal by binding - clearance

27 $$\begin{aligned} \frac{d}{dt}(Q)= cQM2*M2 - wdECMQ*Q*ECM-eF*Q \end{aligned}$$

The rate of change of FS sequestered fibers not part of MP

28 $$\begin{aligned} \frac{d}{dt}(FS)= q*F \end{aligned}$$

A description of the parameters in the model and a table listing parameter values is given in Appendix A.

## Numerical simulations

All numerical simulations were performed using MATLAB R2023a (MathWorks 2023). The system of ordinary differential equations (1)-(28) was solved using the ode15s solver. Parameter values were obtained from biological and experimental literature where available. A detailed description of parameter choices is given in Appendix A. Simulations were run for different time scales in order to study transient dynamics and long-term behavior. Parameter sensitivity analysis was conducted by systematically varying each parameter by ±1% around default values while holding others constant.

**Fig. 2:**
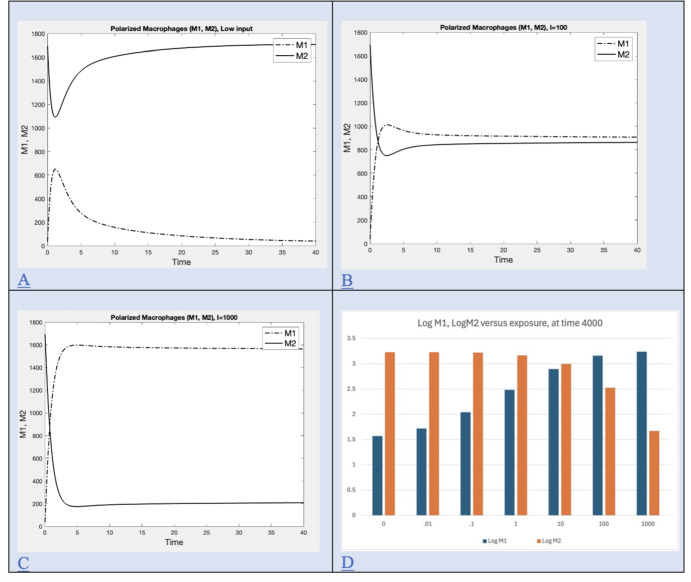
Macrophage polarization in response to exposure. Polarization happens immediately upon exposure. Levels remain changed after exposure ends. A) Early macrophage phenotype (M1, M2) dynamics for low exposure level (I=0.01, t=1095 days), B) Early macrophage phenotype (M1, M2) dynamics for intermediate (I=100, t=1095 days) exposure level, C) Early macrophage phenotype (M1, M2) dynamics for high exposure level (I=1000, t=1095 days), D) Bar graph for long term levels for various exposure rates expressed as $log_{10}$ of M1 and M2 at t=4000

Time series for polarized macrophages (M1, M2) at default values for various time intervals are shown in Figure 2 A, B, C.

**Fig. 3:**
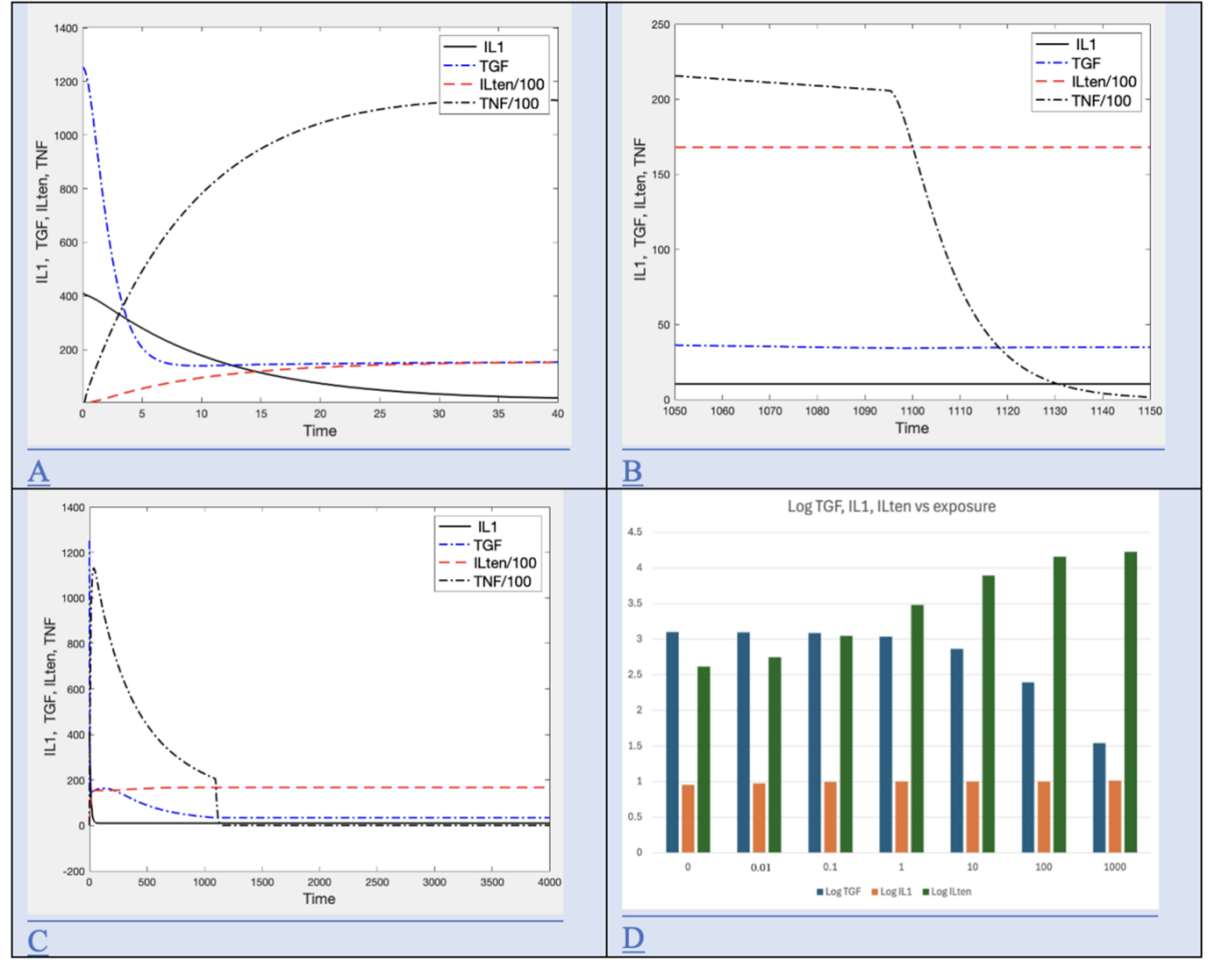
Signal dynamics A) Early cytokine signaling dynamics for high exposure (I=1000, t=40 days) B) Mid cytokine signaling dynamics for high exposure (I=1000, t=1050-1150 days) C) Long term cytokine signaling dynamics for high exposure (I=1000, t=4000 days) D) Bar graph for long term levels of cytokine signals for various exposure rates expressed as $log_{10}$ of TGF, IL1 and ILten at t=4000

Time series for cytokine signals in the model (IL1, ILten, TGF, TNF) at default values for various time intervals are shown in Figure 3 A, B, C. Values for ILten and TNF are divided by 100 in the plot for scaling purposes.

**Fig. 4:**
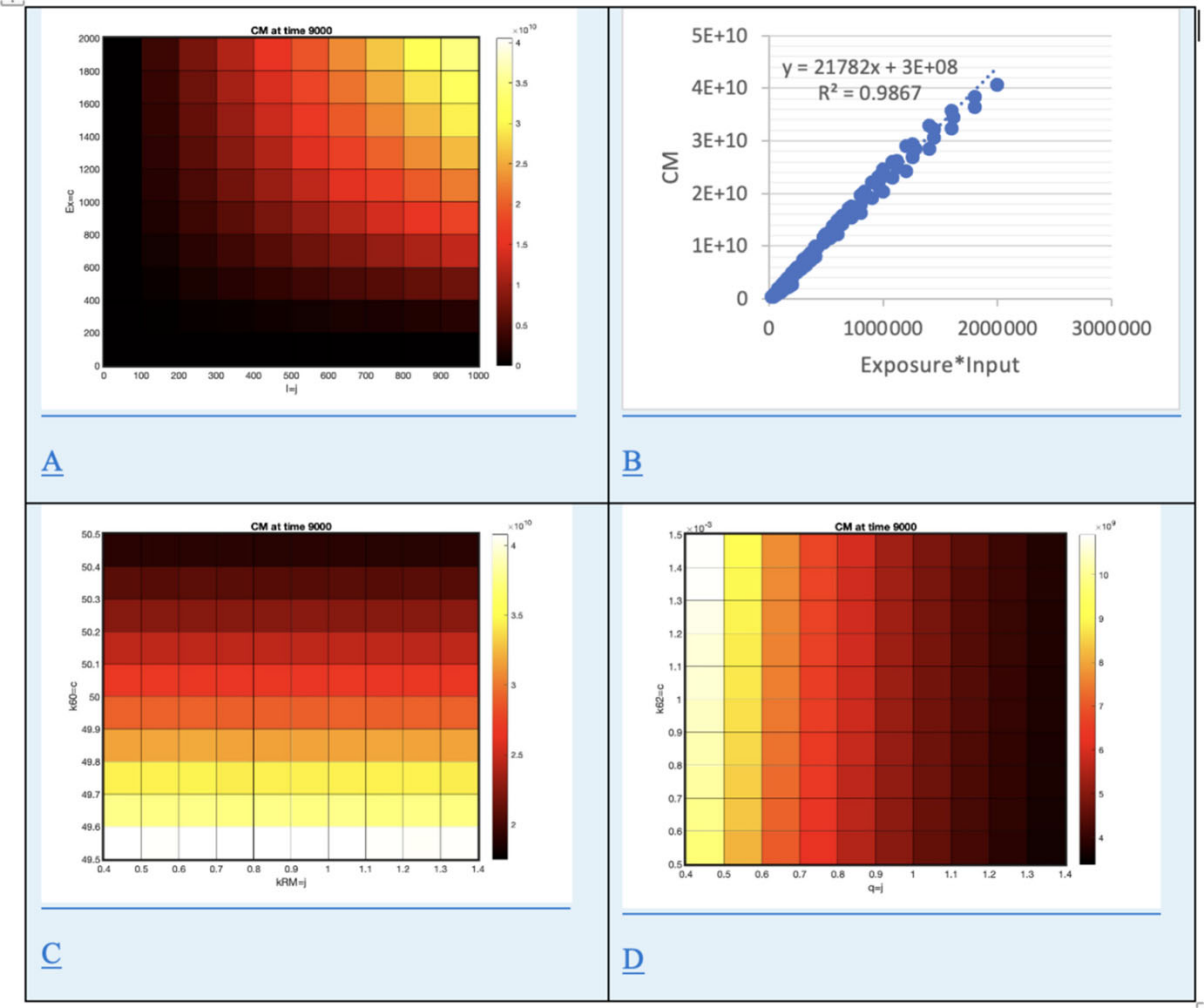
Cancer cell (CM) levels at t=9000 days. A) Heat map for cancer levels (CM) versus exposure level (I) and duration (EX). B) Scatterplot of cancer levels (CM) versus the product of exposure level (I) and duration (EX). C) Heat map for cancer levels (CM) versus macrophage recruitment (kRM) and t-cell carrying capacity (k60). D) Heat map for cancer levels (CM) versus free fiber sequestration rate (q) and incomplete phagocytosis rate (k62)

The relationship of cancer cell population (CM) to exposure level (I) and exposure duration (EX) was computed as heatmaps of cancer levels versus exposure level and duration for two levels of granularity, shown in Figure 4 A,B,D.

**Fig. 5:**
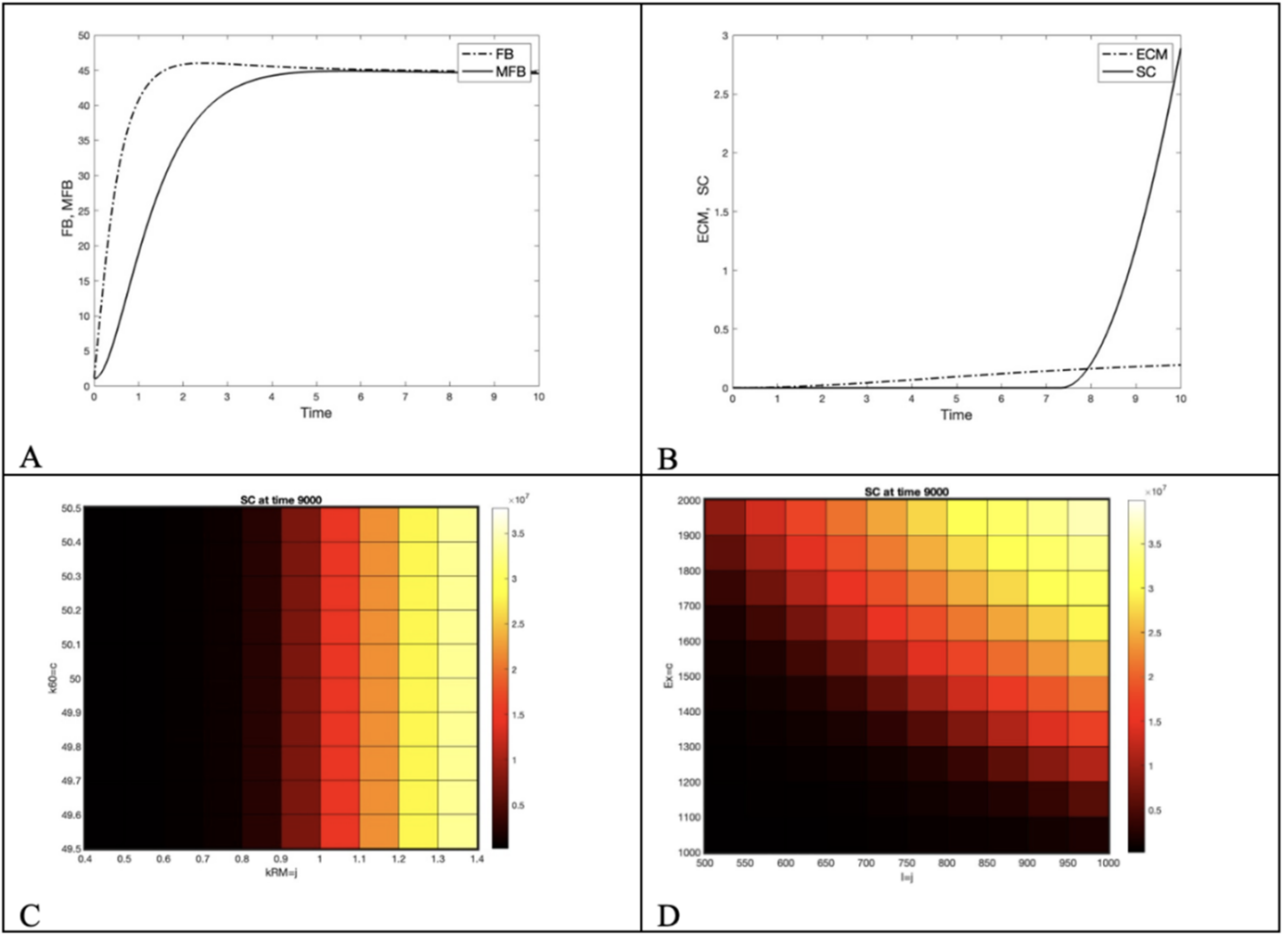
The dynamics of plaque deposition. A) Early fibroblast (FB) and myofibroblast (MFB) dynamics at high exposure level (I=1000, t=10 days), B) Early extracellular matrix (ECM) and plaque (SC) dynamics at high exposure level (I=1000, 10 days) with threshold for deposition (th=.15), C) Heatmap for plaque (SC) deposition at t=9000 versus T-cell capacity (k60) and macrophage recruitment rate (kRM), D) Heatmap for plaque deposition (SC) at t=9000 versus level of exposure (I) and the duration of exposure (EX)

Time series for fibroblasts (FB), myofibroblasts (MFB) and extra-cellular matrix (ECM), at default values for various time intervals, are shown in Figure 5 A, B. A heatmap for plaque (SC) deposition at t=9000 versus T-cell capacity (k60) and macrophage recruitment rate (kRM) is shown in Figure 5 C, and a heatmap for plaque deposition (SC) at t=9000 versus level of exposure (I) and the duration of exposure (EX) is shown in Figure 5 D.

**Fig. 6:**
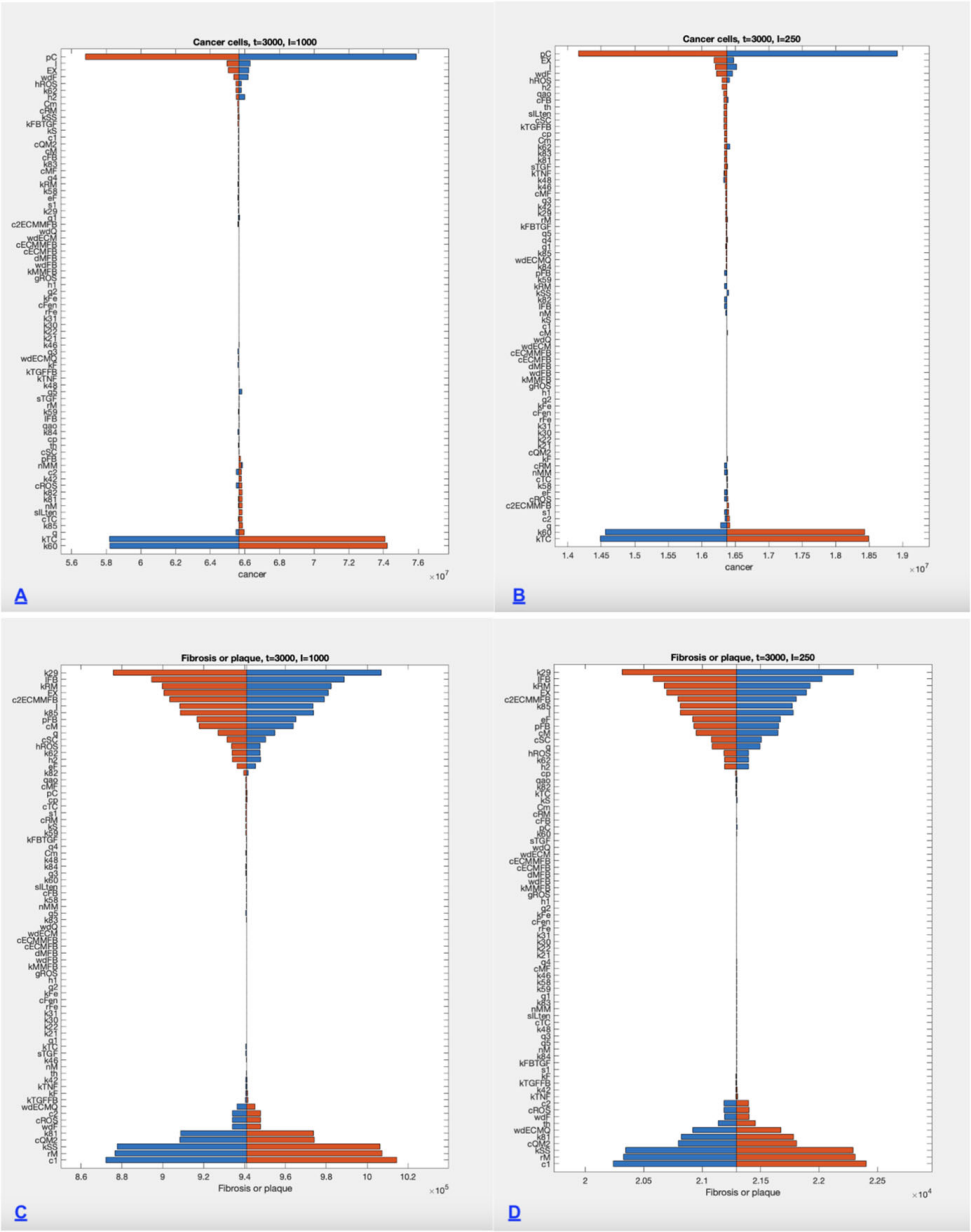
Sensitivity of cancer levels and plaque levels to all model parameters, illustrated with tornado diagrams at two exposure levels. Each parameter in the model was varied separately by one percent. See results section for lists of most influential parameters. A) Sensitivity of cancer level (high exposure I=1000, t=3000) B) Sensitivity of cancer level (lower exposure I=250, t=3000) C) Sensitivity of plaque level (high exposure I=1000, t=3000) D) Sensitivity of plaque level (lower exposure I=250, t=3000)

A local sensitivity analysis was done on the size of the cancer cell population (CM) at time $t=3000$ for both default parameters, low (I=250) and high (I=1000) exposure levels. Each parameter in the model was varied separately by one percent. The results are shown in Figure 6 A,B. A local sensitivity analysis for plaque development (SC) was done for the high exposure default level, shown in Figure 6 C, D. At the low exposure level negligible fibrosis develops.

**Fig. 7:**
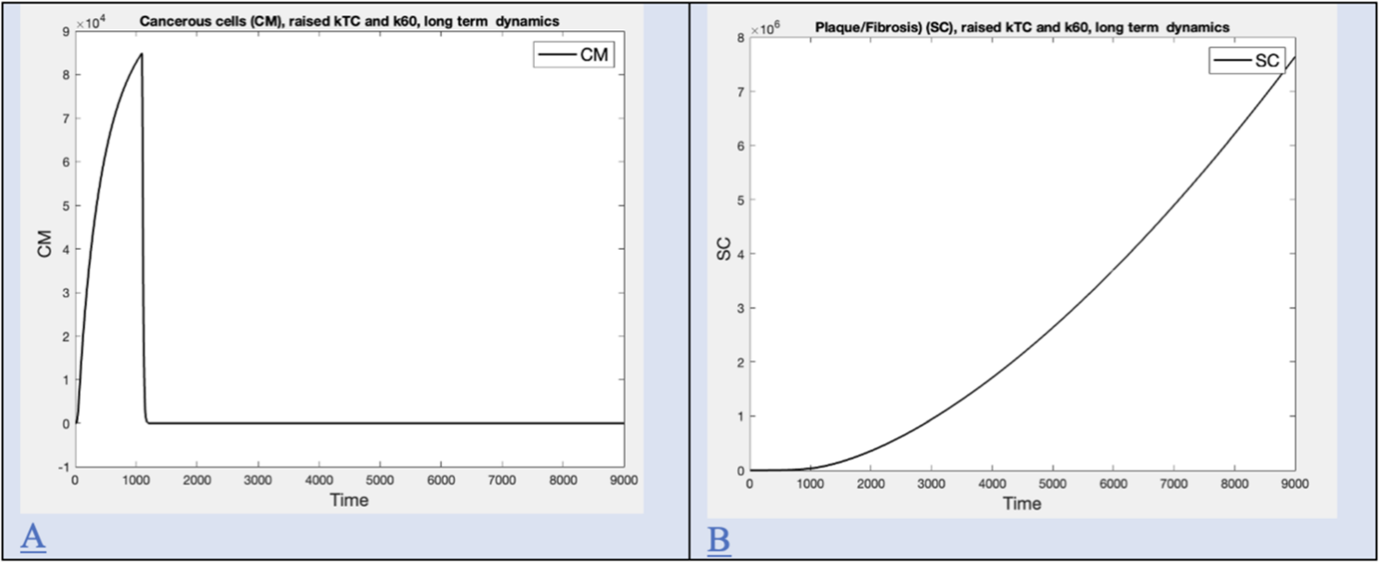
Long term dynamics for plaque/fibrosis with no cancer. A) Long term cancer level (CM) with raised kTC and k60 for high exposure (I=1000, t=9000) B) Long term plaque/fibrosis level (SC) with raised kTC and k60 for high exposure (I=1000, t=9000)

Time series for plaque and cancer are computed by adjusting parameters to enhance the efficiency of T cells in order to illustrate the case where plaque appeared with no mesothelioma and are shown in Figure 7.

**Fig. 8:**
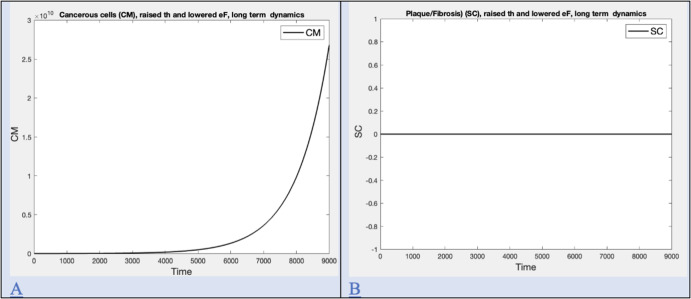
Long term dynamics for cancer with no plaque/fibrosis. A) Long term cancer level (CM) with raised th and lowered eF for high exposure (I=1000, t=9000) B) Long term plaque/fibrosis level (SC) with raised th and lowered eF for high exposure (I=1000, t=9000)

Time series for plaque and cancer are computed by adjusting parameters related to formation of extra-cellular matrix in order to illustrate the case where plaque appeared with no mesothelioma and are shown in Figure 8.

## Results

### Macrophage Dynamics

Macrophage polarization happens immediately upon exposure and levels are observed to stabilize before exposure ends. Exposure increases the level of M1 and reduces that of M2 (see Figure 2 A,B, and C) and does so in a dose dependent manner (see Figure 2 D).

### Signal Dynamics

Signaling responses happen immediately upon exposure and levels are observed to stabilize before exposure ends. Initially, TNF and ILten rise while IL10 and TGF drop (Figure 3 A). When exposure ends (t= 1095 days), the level of TNF drops rapidly, while other levels remain approximately the same (Figure 3 B). TNF subsequently drops to zero while other levels remain constant (Figure 3 C) Long after exposure ends, the level of ILten has permanently increased and the level of TGF has decreased in a dose dependent manner, while IL1 levels remain similar (see Figure 3 D).

### Disease Dynamics

Cancer cells develop and proliferate in the model at a rate that depends on exposure level (*I*) and exposure duration (*EX*) as seen in Figure 4 A. Cancer cell populations (*CM*) are linearly correlated with cumulative exposure ($I*EX$, $R^2=0.9867$) as seen in Figure 4 B. As the population of T cells increases with larger carrying capacity, cancer cell populations decline, while increased macrophage recruitment has little effect, seen in Figure 4 C. In this model, sequestered fibers are not available for macrophage ingestion and thus do not become the ferruginous bodies thought to initiate cancer. Thus as the sequestration rate rises, cancer population declines, but as incomplete phagocytosis rates rise, so does the cancer rate, seen in Figure 4 D.

Plaque, treated in the model as analogous to the scar tissue in wound healing (SC) is the result of signals that recruit fibroblasts (FB) and transition these to myofibroblasts (MFB), seen in its early stage in Figure 5 A. The myofibroblasts deposit extracellular matrix (ECM) in a dynamic process with a dissolving enzyme. When extracellular matrix exceeds a given threshold plaque begins to be deposited, seen in Figure 5 B. Macrophages are responsible for signals, and so an increased recruitment rate for macrophages is correlated with plaque deposition, whereas increased T-cell capacity has negligible effect, as seen in Figure 5 C. Plaque formation rises with both the level of exposure (I) and the duration of exposure (EX), as seen in Figure 5 D.

### Sensitivity Analysis

A local sensitivity analysis was done near both high (I=1000) and mid (I=250) levels of exposure with all other parameters varied near default values. The measured outcomes were cancer and plaque levels at t=3000, and different profiles were observed for these two outcomes.

#### Sensitivity of Cancer levels

Cancer levels (at t=3000) are seen to increase substantially with increased levels of six parameters (pC, I, EX, wdF, hROS, k62 for high exposure, h2) and decreased levels of 2 parameters (k60,kTC) for both high (I=1000) and mid (I=250) levels, shown in Figures 6 A and B. Other parameters had little effect.

Cancer promoting factors include the proliferation rate of cancer cells (pC), exposure level and duration (I, EX), the encounter rate of mesothelial cells with fibers (wdF), production rate of reactive oxygen species by macrophages with incomplete phagocytosis (hROS), and rate of incomplete phagocytosis (k62). Cancer inhibiting factors include increased capacity for recruited T cells (k60), and increased predation rate of T cells on mesothelial cells with DNA damage (kTC).

#### Sensitivity of Plaque deposition

Plaque deposition (at t=3000) is seen to increase substantially with increased levels of k29 (clearance M1 and M2), IFB (FB recruitment rate), kRM (recruitment rate of RM from MO), EX (exposure duration), c2eECMMFB (TGF related ECM production rate), I (exposure level), k85 (Ilten clearance rate), and pFB (TGF related transition rate of FB).

Plaque deposition is seen to increase with decreased levels of c1 (FB, and MFB clearance rate), rM (MO recruitment), kSS (polarization rate), cQM2 (enzyme production rate), k81, (Ilten production rate by M1), wdECMQ (ECM reaction rate), th (threshold for plaque deposition), and wdF (rate of mesothelial cells exposure to fibers). Other parameters had little effect. These responses are shown in Figure 6 C and D.

### Plaque/Fibrosis with no Cancer

We note that plaques (SC) can occur without mesothelioma (CM). Our model reflects this with appropriate choices of parameters to enhance the efficiency of T cells (kTC and K60). Times series are shown in Figure 7 A and B.

### Cancer with no Plaque/Fibrosis

We note that plaque (SC) can occur without mesothelioma (CM), although this is less common. Our model reflects this with appropriate choices of parameters related to formation of extracellular matrix (th, eF). Times series are shown in Figure 8 A and B.

## Discussion

A process based model was developed for mesothelial damage and repair, based on the paradigm of wound healing. Cancer initiation was included as a result of the creation of reactive oxygen species associated to the frustrated phagocytosis of asbestos fibers by macrophages. The model, based on biological understanding, produces realistic asbestos burdens per gram of wet tissue.

The model is consistent with the observation that cancer is slow to develop, with higher levels after a longer duration (Lacourt et al. 2017). The speed of development increases with both exposure levels and duration. The model gives a slightly sublinear response to cumulative exposure (level*duration) under the assumption of equal time since first exposure, consistent with some analyses (Hodgson and Darnton 2000). There appears to be little difference between intense short exposure and moderate long exposure, as long as cumulative exposure remains the same.

The model shows plaque or fibrosis continuing to form after exposure ends. This phenomenon is due to the elevated levels of fibroblasts in the presence of sequestered fibers. These levels are also reflected in changes in the levels of various signaling molecules, which remain elevated or depressed after exposure ends.

### Parameters and Sensitivity

A complex model such as the one used for this study must necessarily include many parameters. When studying a human disease with a long time frame such as mesothelioma, it is not usually possible to measure all quantities directly in the body, nor is it possible to have a time series for a single biomarker that spans the entire time of development. In addition, many parameters reflect processes that vary widely from individual to individual, making any choice of parameters uncertain. This worry is one reason for a sensitivity analysis, done here for two levels of exposure, with respect to both cancer and fibrosis levels at the 3000 day point, shown in Figure 6.

#### Sensitivity of parameters for cancer levels

The sensitivity for cancer levels is shown to depend on very few parameters, all of which have to do with the immediate dynamics of cancer initiation and growth. Cancer growth rate has the largest positive effect. This rate is particularly difficult to estimate in reality, because the disease is not diagnosed until it is very advanced. Mutation rates can be estimated in other contexts, but of course not all mutations lead to cancer. It is not known how much this rate might vary from one individual to the next. Some treatments seek to reduce the growth rate of mesothelioma, but not until the tumor is very advanced. The rate chosen for numerical experiment gives enough cancerous cells in 9000 days to explore the relationship of tumor size to exposure level and duration, seen in Figure 4b. It is this comparative relationship that is informative.

Exposure level and duration also have a large positive effect on tumor size. These are known to vary widely among patients. An autopsy study by Dodson et al (Dodson et al. 2007) considers 11 patients who died of asbestos-related disease. Exposure duration ranged from 5 to over 30 years. In a prior study DeStefano et al. (2021) attempted to use a transport model to estimate exposure levels leading to reported fiber densities, using the result with some success to predict levels in lymph nodes. Exposure levels were also found to vary greatly, unsurprising given that the 11 patients came from different workplace environments.

The parameter k62 is of special interest. This is the rate of incomplete phagocytosis, which leads to ROS production and consequently cancer and was set initially to 0.001, or one fiber in a thousand. It has a positive effect on cancer development, as seen in the sensitivity analysis. The effect is not as big as one would expect, given it is necessary for the onset of cancer. This parameter is related implicitly to particle length because it is thought that incomplete phagocytosis results from fibers too long to engulf. The sensitivity analysis suggests that although some long fibers are necessary to initiate cancer, having more of them might not have a higher impact in the longer term. This makes sense because those semi-ingested fibers cannot be cleared from the tissue and continue to produce ROS.

Improved T-cell dynamics have the largest negative effect. It is known that immune system dynamics differ greatly among individuals. In no study of mesothelioma patients that we found was there an assay of T-cell levels. The sensitivity analysis suggests that the T-cell population might be a useful therapeutic target for mesothelioma.

Many parameters had little to no effect on cancer levels when varied. These parameters are estimated roughly, but are clearly less important to the outcome of the model regarding cancer initiation and development.

#### Sensitivity of parameters for plaque

The deposition of plaque requires interactions among multiple signaling molecules and cell types, in a process adapted from wound healing models. Not surprisingly, many associated parameters have comparably big effects on the final amount of fibrosis, in contrast to the sensitivity of cancer development. It will be easiest to look at the process of fibrosis starting at the endpoint.

Fibrosis and plaque deposition happen because the extra-cellular matrix (ECM) has not been degraded by the enzyme (Q). We see that increased production of ECM (c2ECMMFB) has a large positive effect on final SC, while lowering the threshold for deposition (th), increasing the enzyme production rate (cQM2) and associated reaction rate (wdECMQ), all have a large negative impact on SC.

Extra-cellular matrix is deposited by myofibroblasts, which are transitioned from fibroblasts in the presence of transitional growth factor (TGF). Increasing the rate of fibroblast recruitment (IFB) and the transition rate to myofibroblast (pFB) increases the final SC, while increasing the clearance rate of these cells (c1) reduces final SC.

The degrading enzyme is produced by M2 polarized macrophages. Increasing M2 should reduce the SC deposited. Macrophages are polarized to the M2 form by the presence of IL-ten, which is, in turn, produced by M1 macrophages. Increasing this production rate (k81) results in a decrease in SC, while increasing its clearance rate (k85) increases final SC. Increasing the clearance rate of M2 (k29) has the biggest positive effect on final SC. Rate of mesothelial exposure to fibers resulting in death of the cell (wdF) has a large negative effect on final SC levels. The presence of a dead mesothelial cell requires removal by a macrophage, (RM or M1) and thus a reduction of M1 levels. All of these effects point to the important role of macrophages in this process.

The balance of M1 and M2 is controlled by the pro-inflammatory (p) and anti-inflammatory (p2) cytokines, respectively. The pro-inflammatory cytokines, produced by M1, will also summon fibroblasts. Increasing the circulating monocyte recruitment rate (rM) makes more monocytes available for all functions, yet it has a large negative effect on final SC. Increased recruitment of resident monocytes (kRM) removes these cells from the polarization options and has a large positive effect on final scar tissue. As inflammation, deposition and degradation of ECM are ongoing processes, it might be possible to measure inflammatory markers in short term animal models of asbestosis. Clarifying the parameters associated to this part of the model could yield biomarkers that identify those asbestos-exposed individuals at risk for fibrosis.

Finally, we must note that exposure level (I) and duration (EX) both have large positive effects on final SC levels. This is expected to be the case. However, given the complexity of the processes leading to fibrosis and plaque, it is not at all obvious from model equations that this outcome would occur.

### Model validation

Mesothelioma is a rare disease usually diagnosed in its later stages. Data about this disease comes in the form of autopsy records done years to decades after exposure to asbestos has ended. Fibrosis and plaque are also usually seen in these autopsies. All data is epidemiological, with efforts made to infer relationships between disease and asbestos exposure.

The invisible workings of the immune system during asbestos exposure are short term dynamics that we argue are similar in nature to those of wound healing. Short term dynamics produced by the model may be compared qualitatively to the dynamics of wound healing, developed by Voropaeva and Bayadilov (2020) and parameterized by data describing measured cytokine responses. The discussion of validation is therefore divided into short and long term dynamics.

#### Short term dynamics

Short term signaling dynamics (IL1, ILten, TGF, TNF) have an initial transient response and equilibrate within days or weeks, as seen in Figure 3. These signaling molecules are part of the wound healing model of Voropaeva and Bayadilov (2020), and are seen in their paper to have similar dynamics, with two differences. First, the wound healing dynamics are a bit faster. The mesothelioma model uses rate constants for the cytokines that are on the low end of those estimated in the wound healing model, at least in part for computational reasons, as the model must run for a 20 year time frame. Second, in the mesothelioma model, TNF is released in response to dead mesothelial cells in the model and drops off at the end of the exposure time rather than remaining at the temporary equilibrium seen in Figure 3A. This is to be expected because asbestos exposure is not a short disruption like a wound, but rather an ongoing process for years.

Sterile inflammation (in the absence of infection) is known to occur because of intracellular contents release from dying cells, as is the case with both wound healing and the processes described in this model (Chen and Nuñez 2010). It is also known to occur because of exogenous material such as sequestered fibers (Chen and Nuñez 2010). This process is explicitly included in the model presented here and is the reason for an immune response that continues after exposure ends.

#### Long term dynamics

At its incipient stages a tumor will be too small to detect. If an autopsy finds mesothelioma, that is because it has grown to a detectable size. In order to compare the deterministic model developed here with epidemiological data, one must make the leap of accepting the amount of cancer or fibrosis predicted by the model as indicative of the likelihood of developing visible disease, otherwise described as disease risk.

One interesting feature of mesothelioma is that the risk of developing it increases with the time that has passed since exposure (Berry et al. 2004; Reid et al. 2014). The model mirrors this, with tumor growth continuing after exposure ends.

Risk of cancer is found to rise with higher intensity and duration of exposure (Baris et al. 1987; Comin et al. 1997; Hodgson and Darnton 2000). The model echoes this finding, as seen in Figure 4a. Hodgson and Darnton claim further that in their sample, pleural mesothelioma risk has a slightly sub-linear relation with cumulative exposure (Hodgson and Darnton 2000). Figure 4b shows that the model exhibits a similar pattern. The model also links tumor size to incomplete phagocytosis (k62), seen in Figure 4D at low levels of fiber sequestration (q). Incomplete phagocytosis is hypothesized to be due to larger particle size, which is linked in studies to risk of mesothelioma (Boulanger et al. 2014).

The model predicts a relationship of fibrosis/plaque to both intensity and duration of exposure, seen in Figure 6D. Mossman and Churg point out that asbestosis appears sooner in a patient after higher exposure, consistent with the relationship given by the model (Mossman and Churg 1998). Based on a review of studies, they assert that asbestos is a fiber dose-driven disease but point out that only a fraction of exposed individuals develops this disease, further suggesting that individual variation is responsible (Mossman and Churg 1998). The sensitivity analysis shown in Figure 6 shows that many aspects of the immune system model could be in play. Some of these are now recognized in experimental settings (Dostert et al. 2008). Asbestosis can also occur in the absence of mesothelioma, a possibility that the model duplicates in Figure 7 by increasing the number of available T cells and their efficiency. Mesothelioma is occasionally but rarely found without pleural plaques (Touza et al. 2025), a possiblity the model duplicates in Figure 8 by adjusting parameters related to extra-cellular matrix formation.

### Fiber burden in autopsy tissue

The main quantifiable measure associated to autopsy data is fiber burden. This is measured in a variety of ways and is unlikely to be consistent across studies (Mossman and Churg 1998). Fiber burden is calculated on the pleural tissue as a whole. One would expect the mesothelium to have a lower fiber burden than the lung itself, as fibers enter through the air passages and have opportunities to be removed or get lodged there rather than passing through to the mesothelium. In addition, the example done with the model only has 3 years of exposure, while autopsy subjects often have much longer exposure. Fibers are often distinguished as uncoated or coated. The coated fibers are believed to be the result of incomplete phagocytosis.

Measured in the model at t=2000 after exposure ends, the uncoated fibers (FS) are at $6.15*10^5$ per $10^{10}$ mesothelial cells, and coated fibers (MP) are at 480. If one assumes $10^8$ cells per gram of tissue (Wilson et al. 2003), this translates to about 6,150 uncoated and about 5 coated fibers per wet gram of tissue. Using a conversion factor of 6.56 (Dodson et al. 2007), there would be about 40,344 uncoated and 33 coated fibers per dry gram.

These numbers are within the range of those reported by Dodson et al. (2007) and Kishimoto et al. (2011) but at the very low end of the scale. Mossman and Churg refer to three studies giving median fiber concentrations per dry gram anywhere from 2,200 to over $100*10^6$ (Mossman and Churg 1998). Similar ranges are reported in other studies (Laaksonen et al. 2022; Tuomi et al. 1989; Visonà et al. 2023). These ranges are enormous but at least the model is within range and at the lower end as expected. Not every study reports coated fibers, but the Dodson et al study finds between 0 and 5000 coated fibers per wet gram among his 11 autopsy subjects (Dodson et al. 2007). The number of coated fibers rises with the uncoated fiber count, but not consistently.

## Conclusion

Short term physiological responses to a toxin, such as asbestos fibers, may produce disease in the very long term, making it difficult to monitor the progress of disease in individuals known to have been exposed. Because a disease like mesothelioma or fibrosis does not present symptoms for years or decades, there is necessarily a lack of data about intermediate processes and disease progress.

Modeling a phenomenon dependent on processes of very different time frames is also difficult, but this study shows that it can be done with realistic time frames and biologically reasonable results. Cancer development and plaque deposition are shown to be dependent on total exposure (intensity * duration). This study shows that the short-term process of inflammatory immune response, coupled with the medium duration process of asbestos inhalation and the long-term processes leading to initiation and growth of a tumor, can account for both plaque/fibrosis deposition and the onset of mesothelioma.

The model presented in this study, and its sensitivity analysis, suggest an approach to cancer treatment based on an immune system therapy designed to enhance the effectiveness of T-cell dynamics. It also suggests an approach to monitoring the likelihood of fibrosis development through assays of cytokine levels, which change in the ongoing presence of sequestered fibers. Large scale clinical studies could determine if such assays produce a significant difference in cytokine expression between exposed and unexposed subjects.

The approach in this study would also be useful in understanding the possible effects of exposure to other types of inhaled fibers, such as nanofibers, which may have similar physical properties to asbestos, but different chemical properties.

## Data Availability

MATLAB code to numerically solve the ODE model and to produce the figures is available at https://github.com/aad-314/AsbestosDiseaseInitiation

## Appendix A Model Parameters

A description of the parameters is below, followed by a table with parameter values.

**Equations 1 through 6: macrophage dynamics**

Recruitment ($$rM = 1.2 10^5$$) and clearance (cM = 0.5) of circulating monocytes were chosen to give a steady state of 240,000 monocytes per $$10^6 $$cubic micrometers, equivalent to 240 per microliter, within observed range. Recruitment of macrophages from the circulating monocytes is assumed to be high ($$kRM=.9$$) with low clearance ($$cRM=.0167$$) because the resident macrophages are not circulating. Parameters kSS and kS describe the rate of polarization of macrophages to either M1 or M2. These were taken from Torres et al. (2019), in which a model of inflammation is fit to data. The clearance rate for M1 and M2 ($$k29 = 1$$) is chosen to give the polarized macrophages one day of residence. The real rate is not known. A small percent of fibers taken up by macrophages result in incomplete phagocytosis ($$k62 = 0.001$$). This percent depends on the percent of fibers that are too long to be completely engulfed and removed, which depends, in turn, on the particular type of asbestos inhaled and is not, in general, known.

**Equations 7 to 10: cytokine production**

The system of cytokine and other signal interactions given in this study was simplified from that of Voropaeva and Bayadilov of wound healing (Voropaeva and Bayadilov 2020), by combining the overall effects of pro-inflammatory cytokines (represented by IL1) and combining the anti-inflammatory cytokines (represented in the model by Ilten). As a result, there is not a strict correspondence in parameters. Clearance rates for cytokines ($k85= .1$) were chosen in the range reported (.058 to .193) in Voropaeva and Bayadilov. Maximum production rates of cytokines (*k*58, *k*81, *k*83) and half saturation rates (*k*59,*k*82, and *k*84) were chosen within the rather large reported values (0 to 254) in Voropaeva and Bayadilov, where smaller values predominate. All of these except k58 were set to 1. However, to ensure some kind of immune response, the pro-inflammatory cytokines need to be produced at a higher level, hence $k58=10$. Half saturation rates for cytokines (*k*59, *k*82, *k*84) were also set to 1, again well within the reported values. A similar analysis for TNF resulted in production rate $cp=1$, and half saturation rate $kTNF = 1$. The reported clearance rate for TNF seemed high, so the cytokine clearance rate was used ($k42=0.1$). The dynamics used for TGF in the model here were linear and rates could be taken directly from Voropaeva and Bayadilov ($k46=0.512$, $k48=0.693$).

**Equations 11 to 22: dynamics of mesothelial cells and DNA damage**

It is known that mesothelial cells ingest asbestos rapidly and die in a dose dependent manner (Broaddus et al. 1996). This observation would require $c2=1$. However, these observations were made in a very high dose situation *in vitro* and cells were dead within the day. It is not known what the rate constants would be in real situations. Because it is not possible to track the progress of human mesothelioma during its multi-decade progression, many parameters describing long term processes can only be characterized by their speed relative to other, known processes. In particular, the production rate of ROS in vivo is not known and the resulting DNA damage is still a hypothesis. Units are unspecified but the duration in the system is taken to be 1 day ($hROS=cROS=1$).

Mesothelial cells can take a mesenchymal form and proliferate in response to signals, but the dose response is unknown. Relative population size of these two forms has not been measured but the mesenchymal form is relatively rare. For this reason, the transition from M to MM is slow ($q1=0.0001$, $s1=100$). Proliferation is also possible ($q3=0.01$) but results in the non-mesenchymal form and only up to a preset carrying capacity, as this is the mechanism of repair of mesothelial tissue.

Finally, cancer processes are slow for mesothelioma, with visible symptoms often only decades after exposure. The rate of production and clearance ($hROS = cROS =1$) are relatively fast but the onset and growth of cancer ($nM= nMM=0.0001,pC=0.006$ ) in response to ROS is very slow. The response of T cells to both signals and the presence of the cancer itself is relatively fast, in line with other signaling and macrophage dynamics, although clearance is relatively slow ($q4=sTGF=sILten=1$, $cTC=0.0001$) as T cells are relatively long lived.

Fiber input (I), exposure duration (EX), and sequestration rate (q) all depend on intensity of exposure, duration of exposure, and properties of the fiber. These will be different for every patient and are varied in this study. The fiber sequestration rate (*q*) is taken to be higher than the one computed in DeStefano et al. (2021) because the mesothelium is assumed to be a denser tissue than generalized lung tissue.

**Equations 23 to 28: dynamics of fibroblast arrival, deposition of extracellular matrix, and formation of plaque or fibrosis**

The dynamics of fibroblasts, myofibroblasts and extra-cellular matrix (ECM) are modeled after (Hao et al. 2015) with some adjustments. Hao et al’s model produces reasonable dynamics for fibrosis deposition for a time frame up to 300 days. However, their parameters include a very large clearance rate for the degrading enzyme (eF) (on the order of $10^7$) and a very small half saturation constant for deposition of ECM (*c*2*ECMMFB*) (on the order of $10^{-30}$). The clearance rate is not biologically reasonable, as is clear by comparison with other clearance rates of small molecules. Both of these rates also cause numerical issues when the model is run for 20 years instead of 300 days. The clearance rate, *wdECMQ*, was adjusted to 1, and the half saturation rate*kFBTGF* was also adjusted to 1. Other rates associated to fibroblasts and extracellular matrix (*c*1, *c*2*ECMMFB*, *kTGFFB*, *th*, *pFB*, *IFB*, *cSC*, *cQM*2, *eF* and *q*) were adjusted until the short-term dynamics of fibrosis and fibroblasts resembled those of Hao *et al*.

**Table:**

Parameter	**Value**	**Description**	**Equation #**	**Reference**
kSS	21	Influx rate for polarization A	A,2,5,6	Torres
kS	5.156	Influx rate for polarization A	A,2,5,6,p,p2	Torres
kRM	0.9	Recruitment rate from monocytes	1,2	
kF	0.001	MD ingestion rate	1,3,5,15	
qao	0.01	MD ingestion half saturation	1, 3, 5, 15	
cRM	0.0167	Clearance rate for RM	1	DeStefano
rM	1.2e5	Normal recruitment for MO	2	Torres
cM	0.5	Clearance for MO	2	Torres
cMF	1	MF clearance rate	3,4	
k62	0.001	percent with incomplete phagocytosis	3,4	
k29	1	Clearance rate for M1 and M2	5,6	
k46	0.512	TGF production rate by M2	7	Voropaeva
k48	0.693	TGF clearance rate	7	Voropaeva
cp	1	TNF production rate by M1, M2, and RM	8	
kTNF	1	TNF production half saturation rate	8	
k42	0.1	TNF removal rate	8	
k58	10	IL-1 production by M1	9	Voropaeva
k59	1	IL-1 production dependence on ILten	9	Voropaeva
k85	0.1	IL-1, ILten clearance rate	9,10	Voropaeva
k81	1	IL-10 production by M1	10	Voropaeva
k82	1	IL-10 TGF half saturation rate (M!)	10	Voropaeva
k83	1	IL-10 production by M2	10	Voropaeva
k84	1	IL-10 TGF half saturation rate (M2)	10	Voropaeva
wdF	1	fiber proximity by mesothelial cells	11,12,13,14	
q1	0.0001	M to MM conversion rate	11,12	
s1	100	TGF half saturation parameter	11,12	
q3	0.01	MM, MMNF reproduction rate	11	
Cm	1e10	M carrying capacity	11	
cFB	10	MM to M conversion rate	11, 12	
c2	1	Death rate of MNF, MMNF by fiber uptake	13,14,15,19	
h2	1	DNA damage rate	13,14,16,17	
kTC	0.0001	rate of T-cell removal of MDNA, MMDNA, CM	16,17,18	
nM	0.0001	Cancer onset (M type)	16,18	
nMM	0.0001	Cancer onset (MM type)	17,18	
pC	0.006	Cancer cell proliferation rate	18	
I	1000	Exposure input level (e.g., asbestos)	19	
EX	1095	Exposure duration in days (3 yrs)	19	
q	0.1	fiber sequestration rate	19,28	
hROS	1	ROS production rate	20	
cROS	1	ROS clearance rate	20	
q4	1	T-cell arrival rate due to cytokines	22	
sTGF	1	Scaling for TGF signaling	22	
sILten	1	Scaling for IL-10 signaling	22

**Table:**

Parameter	**Value**	**Description**	**Equation #**	**Reference**
q5	0.1	T-cell arrival rate due to cancer presence	22	
k60	50	T-cell carrying capacity	22	
cTC	0.0001	T-cell clearance rate	22	
IFB	100	fibroblast recruitment rate from cytokines	23	Hao
pFB	1	additional recruitment due to TGF	23,24	Hao
kTGFFB	1	half saturation for TGF	23,24	Hao
c1	1	fibroblast clearance rate	23,24	
c2ECMMFB	0.001	TGF related ECM production rate	25	Hao
kFBTGF	0.1	half saturation for TGF related ECM production	25	Hao
wdECMQ	1	ECM decay/enzyme removal by uptake of Q	25,27	Hao
cSC	1	SC deposition rate	26	
th	0.15	Damage threshold for SC deposition	26	Hao
cQM2	0.001	enzyme production rate	27	Hao
eF	1	enzyme clearance rate	27	

## Declarations

### Materials availability

n/a

### Code availability

The code is available on GitHub.
